# Ethyl 1-(butan-2-yl)-2-(2-meth­oxy­phen­yl)-1*H*-benzimidazole-5-carboxyl­ate

**DOI:** 10.1107/S1600536811046095

**Published:** 2011-11-09

**Authors:** Natarajan Arumugam, Nurziana Ngah, Hasnah Osman, Aisyah Saad Abdul Rahim

**Affiliations:** a School of Pharmaceutical Sciences, Universiti Sains Malaysia, 11800 USM Penang, Malaysia; b Kulliyyah of Science, International Islamic University Malaysia, Bandar Indera Mahkota, 25200 Kuantan, Pahang, Malaysia; c School of Chemical Sciences, Universiti Sains Malaysia, 11800 USM Penang, Malaysia

## Abstract

In the title compound, C_21_H_24_N_2_O_3_, the mean planes of the benzene ring and the benzimidazole ring system form a dihedral angle of 69.94 (7)°. The ethyl group atoms of the ethano­ate fragment are disordered over two sets of sites, with refined occupancies of 0.742 (6) and 0.258 (6). In the crystal, there are weak C—H⋯N hydrogen bonds which connect mol­ecules into chains along the *b* axis. A weak inter­molecular C—H⋯π inter­action is also observed.

## Related literature

For the synthesis and a closely related structure, see: Arumugam *et al.* (2010 ▶). For background to microwave chemistry, see: Kappe & Dallinger (2006 ▶); Hamzah *et al.* (2011 ▶). For the synthesis of benzimidazole derivatives and their applications, see: Wang *et al.* (2011 ▶); VanVliet *et al.* (2005 ▶); Loupy (2002 ▶); Santagada *et al.* (2001 ▶); Nicolaou *et al.* (2000 ▶); Evans *et al.* (1988 ▶). For standard bond lengths, see: Allen *et al.* (1987 ▶).
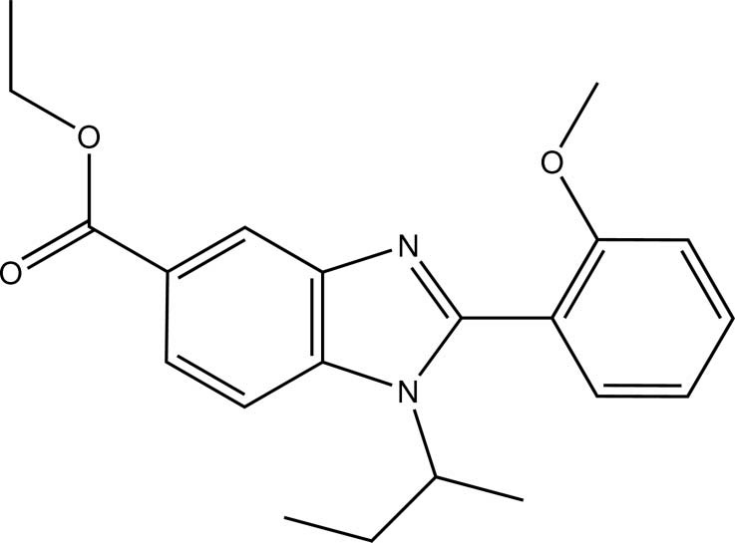


## Experimental

### 

#### Crystal data

C_21_H_24_N_2_O_3_
*M* *_r_* = 352.42
Monoclinic, 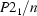

*a* = 10.6746 (3) Å
*b* = 12.3344 (4) Å
*c* = 15.6158 (5) Å
β = 106.901 (1)°
*V* = 1967.25 (11) Å^3^
*Z* = 4
Mo *K*α radiation
μ = 0.08 mm^−1^
*T* = 296 K
0.52 × 0.44 × 0.32 mm

#### Data collection

Bruker SMART APEXII CCD area-detector diffractometer
Absorption correction: multi-scan (*SADABS*; Bruker, 2009 ▶) *T*_min_ = 0.959, *T*_max_ = 0.975
17688 measured reflections
3475 independent reflections
2712 reflections with *I* > 2σ(*I*)
*R*_int_ = 0.025

#### Refinement

*R*[*F* ^2^ > 2σ(*F* ^2^)] = 0.045
*wR*(*F* ^2^) = 0.122
*S* = 1.05
3475 reflections
245 parameters
3 restraints
H-atom parameters constrained
Δρ_max_ = 0.35 e Å^−3^
Δρ_min_ = −0.18 e Å^−3^

### 

Data collection: *APEX2* (Bruker, 2009 ▶); cell refinement: *SAINT* (Bruker, 2009 ▶); data reduction: *SAINT*; program(s) used to solve structure: *SHELXTL* (Sheldrick, 2008 ▶); program(s) used to refine structure: *SHELXTL*; molecular graphics: *SHELXTL*; software used to prepare material for publication: *SHELXTL* and *PLATON* (Spek, 2009 ▶).

## Supplementary Material

Crystal structure: contains datablock(s) global, I. DOI: 10.1107/S1600536811046095/lh5357sup1.cif

Structure factors: contains datablock(s) I. DOI: 10.1107/S1600536811046095/lh5357Isup2.hkl

Supplementary material file. DOI: 10.1107/S1600536811046095/lh5357Isup3.cml

Additional supplementary materials:  crystallographic information; 3D view; checkCIF report

## Figures and Tables

**Table 1:** Hydrogen-bond geometry (Å, °) *Cg* is the centroid of the N1/N2/C1/C2/C7 ring.

*D*—H⋯*A*	*D*—H	H⋯*A*	*D*⋯*A*	*D*—H⋯*A*
C12—H12⋯N1^i^	0.93	2.56	3.471 (2)	165
C20*A*—H20*C*⋯*Cg*^ii^	0.97	2.90	3.71 (4)	141

Symmetry codes: (i) 
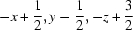
; (ii) 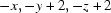.

**Fig. 1.**
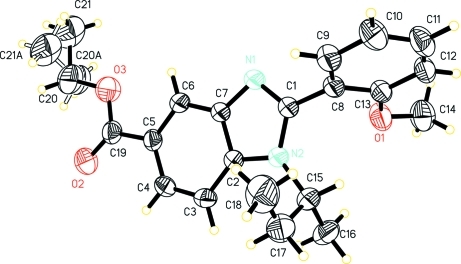
The molecular structure of (I) with displacement ellipsods are drawn at the 40% probability level. Both disordered component are shown, atoms label with suffix A correspond to minor disorder component.

**Fig. 2.**
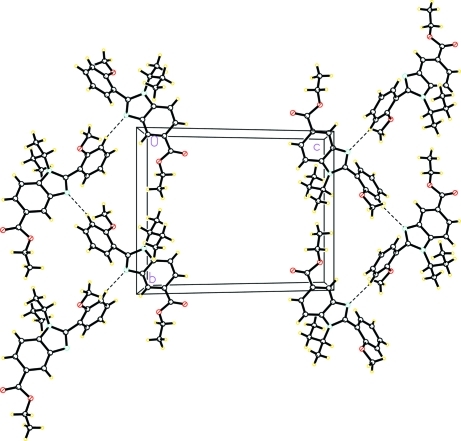
The molecular packing of (I) viewed along the a axis.

## supplementary crystallographic information

### Comment

Microwave-assisted synthesis of heterocycles proves to be an invaluable technology in the fields of medicinal chemistry and drug discovery (Kappe & Dallinger, 2006). The utility of high-speed microwave chemistry is evident from the reported synthesis of privileged structures (Evans *et al.*, 1988; Nicolaou *et al.*, 2000) such as benzodiazepine (Santagada *et al.*, 2001), indoles (Loupy, 2002) and benzimidazoles (Wang *et al.*, 2011; VanVliet *et al.*, 2005). As a part of our on-going work in benzimidazole synthesis under microwave conditions (Hamzah *et al.*, 2011), we present herein the X-ray crystal structure of the title compound.

The molecular structure of the title compound (Fig. 1) is similiar to the previously reported ethyl 1-*sec*-butyl-2-(4-methoxyphenyl) -1*H*-benzimidazole-5-carboxylate (Arumugam *et al.*, 2010) in that only the position of the methoxy group is different. The benzene [C8—C13] ring and benzimidazole ring system [N1/N2/C1-C7] are essentially planar with maximum deviation of 0.050 (1)Å for atom N2. The mean-planes of the rings form a dihedral angle of 69.94 (7)°. The bond lengths (Allen *et al.*, 1987) and angles aree in the normal ranges and comparable to those in *para*-methoxy derivative. The ethyl atoms (C20 & C21) of ethanoate fragment are disordered over two positions with refined site occupancies of 0.742 (6) and 0.258 (6). In the crystal, a C12—H12···N1^i^ hydrogen bond connects molecules to form a *zigzag* chain propagating along the *b* axis (Fig. 2). An weak intermolecular C20A—H20C···*Cg*^ii^ (minor component of disorder) interaction is also observed; *Cg*1 is the centroid of N1/N2/C1/C2/C7.

### Experimental

The title compound was prepared according to our previous procedure (Arumugam *et al.*, 2010). A solution of the *sec*-butyl phenylene diamine (1.0 mmol) and sodium bisulfite adduct of 2-methoxybenzaldehyde (3.5 mmol) in DMF was heated under focused microwave conditions at 403K for 2 minutes. The reaction mixture was diluted in EtOAc (20 ml) and washed with H_2_O (20 ml). The organic layer was pooled together, dried over Na_2_SO_4_, and then removed *in vacuo*. Recrystallization with ethyl acetate gave the title compound as colourless crystals.

### Refinement

All atoms were position geometrically and refined using a riding model, with C—H = 0.93–0.97 Å and *U*_iso_(H)= 1.2 or 1.5*U*_eq_(C). The atoms C20 and C21 are disordered over two sites with site occupancies of 0.742 (6) and 0.258 (2). A rigid body restraint (DELU in SHELXL (Sheldrick, 2008)) was applied for atoms C17 and C18. A rotating group model was applied to the non-disordered methyl groups.

### Crystal data

**Table:**

C_21_H_24_N_2_O_3_	*F*(000) = 752
*M**_r_* = 352.42	*D*_x_ = 1.190 Mg m^−^^3^
Monoclinic, *P*2_1_/*n*	Mo *K*α radiation, λ = 0.71073 Å
Hall symbol: -P 2yn	Cell parameters from 8039 reflections
*a* = 10.6746 (3) Å	θ = 2.1–25.0°
*b* = 12.3344 (4) Å	µ = 0.08 mm^−^^1^
*c* = 15.6158 (5) Å	*T* = 296 K
β = 106.901 (1)°	Block, colourless
*V* = 1967.25 (11) Å^3^	0.52 × 0.44 × 0.32 mm
*Z* = 4	

### Data collection

**Table:**

Bruker SMART APEXII CCD area-detector diffractometer	3475 independent reflections
Radiation source: fine-focus sealed tube	2712 reflections with *I* > 2σ(*I*)
graphite	*R*_int_ = 0.025
Detector resolution: 83.66 pixels mm^-1^	θ_max_ = 25.0°, θ_min_ = 2.1°
φ and ω scan	*h* = −11→12
Absorption correction: multi-scan (*SADABS*; Bruker, 2009)	*k* = −11→14
*T*_min_ = 0.959, *T*_max_ = 0.975	*l* = −18→17
17688 measured reflections	

### Refinement

**Table:**

Refinement on *F*^2^	Primary atom site location: structure-invariant direct methods
Least-squares matrix: full	Secondary atom site location: difference Fourier map
*R*[*F*^2^ > 2σ(*F*^2^)] = 0.045	Hydrogen site location: inferred from neighbouring sites
*wR*(*F*^2^) = 0.122	H-atom parameters constrained
*S* = 1.05	*w* = 1/[σ^2^(*F*_o_^2^) + (0.0526*P*)^2^ + 0.5324*P*] where *P* = (*F*_o_^2^ + 2*F*_c_^2^)/3
3475 reflections	(Δ/σ)_max_ < 0.001
245 parameters	Δρ_max_ = 0.35 e Å^−^^3^
3 restraints	Δρ_min_ = −0.18 e Å^−^^3^

### Special details

**Table:**

Geometry. All e.s.d.'s (except the e.s.d. in the dihedral angle between two l.s. planes) are estimated using the full covariance matrix. The cell e.s.d.'s are taken into account individually in the estimation of e.s.d.'s in distances, angles and torsion angles; correlations between e.s.d.'s in cell parameters are only used when they are defined by crystal symmetry. An approximate (isotropic) treatment of cell e.s.d.'s is used for estimating e.s.d.'s involving l.s. planes.
Refinement. Refinement of *F*^2^ against ALL reflections. The weighted *R*-factor *wR* and goodness of fit *S* are based on *F*^2^, conventional *R*-factors *R* are based on *F*, with *F* set to zero for negative *F*^2^. The threshold expression of *F*^2^ > σ(*F*^2^) is used only for calculating *R*-factors(gt) *etc*. and is not relevant to the choice of reflections for refinement. *R*-factors based on *F*^2^ are statistically about twice as large as those based on *F*, and *R*- factors based on ALL data will be even larger.

### Fractional atomic coordinates and isotropic or equivalent isotropic displacement parameters (Å^2^)

**Table:**

	*x*	*y*	*z*	*U*_iso_*/*U*_eq_	Occ. (<1)
O1	0.22019 (14)	0.60261 (11)	0.83252 (9)	0.0694 (4)	
O2	0.16647 (18)	1.12078 (12)	1.21044 (10)	0.0894 (5)	
O3	0.10964 (19)	1.17279 (12)	1.06857 (10)	0.0917 (5)	
N1	0.29480 (16)	0.88252 (11)	0.90695 (9)	0.0562 (4)	
N2	0.36352 (14)	0.74369 (11)	1.00207 (8)	0.0475 (3)	
C1	0.34617 (16)	0.78558 (13)	0.91776 (10)	0.0467 (4)	
C2	0.32104 (16)	0.82294 (12)	1.04995 (10)	0.0445 (4)	
C3	0.31600 (18)	0.82949 (14)	1.13790 (11)	0.0551 (5)	
H3	0.3450	0.7729	1.1781	0.066*	
C4	0.26638 (18)	0.92316 (14)	1.16254 (11)	0.0542 (4)	
H4	0.2638	0.9304	1.2213	0.065*	
C5	0.21963 (17)	1.00806 (13)	1.10246 (11)	0.0496 (4)	
C6	0.22494 (19)	1.00099 (13)	1.01515 (11)	0.0540 (5)	
H6	0.1942	1.0572	0.9748	0.065*	
C7	0.27727 (17)	0.90788 (13)	0.98941 (10)	0.0473 (4)	
C8	0.38804 (17)	0.72852 (14)	0.84697 (10)	0.0498 (4)	
C9	0.49117 (19)	0.76994 (16)	0.82051 (12)	0.0606 (5)	
H9	0.5345	0.8316	0.8484	0.073*	
C10	0.5308 (2)	0.72096 (19)	0.75319 (13)	0.0711 (6)	
H10	0.6003	0.7493	0.7358	0.085*	
C11	0.4666 (2)	0.63017 (18)	0.71234 (13)	0.0694 (6)	
H11	0.4939	0.5966	0.6675	0.083*	
C12	0.3627 (2)	0.58773 (16)	0.73630 (11)	0.0608 (5)	
H12	0.3196	0.5264	0.7076	0.073*	
C13	0.32254 (18)	0.63726 (14)	0.80372 (11)	0.0517 (4)	
C14	0.1359 (2)	0.52171 (19)	0.78162 (15)	0.0819 (6)	
H14A	0.0981	0.5473	0.7216	0.123*	
H14B	0.0675	0.5063	0.8084	0.123*	
H14C	0.1852	0.4569	0.7805	0.123*	
C15	0.42814 (19)	0.63962 (14)	1.03579 (12)	0.0601 (5)	
H15	0.4483	0.6042	0.9852	0.072*	
C16	0.3357 (2)	0.56259 (16)	1.06687 (15)	0.0782 (6)	
H16A	0.3108	0.5957	1.1151	0.117*	
H16B	0.3800	0.4955	1.0869	0.117*	
H16C	0.2589	0.5488	1.0179	0.117*	
C17	0.5563 (2)	0.65788 (19)	1.10606 (15)	0.0816 (6)	
H17A	0.5395	0.6900	1.1583	0.098*	
H17B	0.5984	0.5884	1.1238	0.098*	
C18	0.6475 (2)	0.7294 (2)	1.07565 (18)	0.1013 (8)	
H18A	0.6583	0.7017	1.0208	0.152*	
H18B	0.7308	0.7312	1.1206	0.152*	
H18C	0.6120	0.8014	1.0660	0.152*	
C19	0.1636 (2)	1.10442 (15)	1.13428 (13)	0.0599 (5)	
C20	0.0550 (11)	1.2722 (4)	1.0931 (10)	0.103 (2)	0.742 (6)
H20A	−0.0207	1.2560	1.1134	0.124*	0.742 (6)
H20B	0.1196	1.3091	1.1409	0.124*	0.742 (6)
C21	0.0176 (5)	1.3391 (3)	1.0141 (4)	0.1145 (15)	0.742 (6)
H21A	−0.0225	1.4045	1.0269	0.172*	0.742 (6)
H21B	−0.0436	1.3005	0.9667	0.172*	0.742 (6)
H21C	0.0938	1.3571	0.9963	0.172*	0.742 (6)
C21A	0.0967 (16)	1.3540 (11)	1.0711 (11)	0.1145 (15)	0.258 (6)
H21D	0.0569	1.4175	1.0873	0.172*	0.258 (6)
H21E	0.0936	1.3581	1.0091	0.172*	0.258 (6)
H21F	0.1863	1.3495	1.1073	0.172*	0.258 (6)
C20A	0.027 (4)	1.2591 (10)	1.086 (3)	0.103 (2)	0.258 (6)
H20C	−0.0605	1.2561	1.0439	0.124*	0.258 (6)
H20D	0.0210	1.2562	1.1463	0.124*	0.258 (6)

### Atomic displacement parameters (Å^2^)

**Table:**

	*U*^11^	*U*^22^	*U*^33^	*U*^12^	*U*^13^	*U*^23^
O1	0.0819 (9)	0.0691 (9)	0.0610 (8)	−0.0156 (7)	0.0268 (7)	−0.0184 (6)
O2	0.1453 (15)	0.0707 (10)	0.0661 (9)	0.0110 (9)	0.0526 (10)	−0.0165 (7)
O3	0.1471 (15)	0.0615 (9)	0.0743 (10)	0.0397 (9)	0.0445 (10)	−0.0029 (8)
N1	0.0877 (11)	0.0440 (8)	0.0398 (8)	0.0137 (7)	0.0233 (7)	0.0026 (6)
N2	0.0624 (9)	0.0405 (7)	0.0401 (7)	0.0093 (6)	0.0158 (6)	0.0030 (6)
C1	0.0582 (10)	0.0426 (9)	0.0392 (9)	0.0031 (8)	0.0140 (7)	−0.0008 (7)
C2	0.0558 (10)	0.0399 (9)	0.0390 (9)	0.0005 (7)	0.0158 (7)	0.0000 (7)
C3	0.0745 (12)	0.0514 (10)	0.0420 (9)	0.0053 (9)	0.0212 (8)	0.0077 (8)
C4	0.0724 (12)	0.0558 (11)	0.0394 (9)	−0.0028 (9)	0.0243 (8)	−0.0021 (8)
C5	0.0639 (11)	0.0422 (9)	0.0473 (10)	−0.0050 (8)	0.0233 (8)	−0.0073 (7)
C6	0.0795 (12)	0.0391 (9)	0.0451 (10)	0.0076 (8)	0.0207 (9)	0.0020 (7)
C7	0.0662 (11)	0.0398 (9)	0.0377 (9)	0.0034 (8)	0.0179 (8)	−0.0004 (7)
C8	0.0630 (11)	0.0465 (10)	0.0396 (9)	0.0106 (8)	0.0146 (8)	−0.0004 (7)
C9	0.0673 (12)	0.0607 (12)	0.0557 (11)	0.0019 (9)	0.0206 (9)	−0.0064 (9)
C10	0.0667 (12)	0.0876 (15)	0.0654 (12)	0.0048 (11)	0.0291 (10)	−0.0104 (11)
C11	0.0724 (13)	0.0841 (15)	0.0542 (11)	0.0195 (11)	0.0224 (10)	−0.0142 (10)
C12	0.0739 (13)	0.0579 (11)	0.0455 (10)	0.0130 (10)	0.0093 (9)	−0.0108 (8)
C13	0.0608 (11)	0.0507 (10)	0.0417 (9)	0.0078 (8)	0.0118 (8)	−0.0020 (8)
C14	0.0747 (14)	0.0818 (15)	0.0828 (15)	−0.0122 (12)	0.0129 (11)	−0.0203 (12)
C15	0.0793 (13)	0.0460 (10)	0.0529 (11)	0.0168 (9)	0.0158 (9)	0.0044 (8)
C16	0.1111 (18)	0.0497 (11)	0.0806 (14)	0.0041 (11)	0.0386 (13)	0.0131 (10)
C17	0.0878 (15)	0.0715 (14)	0.0733 (14)	0.0173 (11)	0.0039 (11)	0.0000 (11)
C18	0.0757 (16)	0.101 (2)	0.111 (2)	−0.0132 (13)	0.0018 (13)	−0.0121 (15)
C19	0.0810 (13)	0.0475 (10)	0.0584 (12)	−0.0050 (9)	0.0315 (10)	−0.0091 (9)
C20	0.153 (6)	0.058 (2)	0.108 (4)	0.034 (3)	0.053 (5)	−0.011 (2)
C21	0.117 (4)	0.081 (2)	0.142 (4)	0.041 (3)	0.031 (3)	0.006 (3)
C21A	0.117 (4)	0.081 (2)	0.142 (4)	0.041 (3)	0.031 (3)	0.006 (3)
C20A	0.153 (6)	0.058 (2)	0.108 (4)	0.034 (3)	0.053 (5)	−0.011 (2)

### Geometric parameters (Å, °)

**Table:**

O1—C13	1.365 (2)	C12—C13	1.388 (2)
O1—C14	1.422 (2)	C12—H12	0.9300
O2—C19	1.198 (2)	C14—H14A	0.9600
O3—C19	1.324 (2)	C14—H14B	0.9600
O3—C20A	1.456 (5)	C14—H14C	0.9600
O3—C20	1.456 (4)	C15—C17	1.501 (3)
N1—C1	1.306 (2)	C15—C16	1.546 (3)
N1—C7	1.390 (2)	C15—H15	0.9800
N2—C1	1.376 (2)	C16—H16A	0.9600
N2—C2	1.385 (2)	C16—H16B	0.9600
N2—C15	1.480 (2)	C16—H16C	0.9600
C1—C8	1.485 (2)	C17—C18	1.489 (3)
C2—C3	1.392 (2)	C17—H17A	0.9700
C2—C7	1.397 (2)	C17—H17B	0.9700
C3—C4	1.372 (2)	C18—H18A	0.9600
C3—H3	0.9300	C18—H18B	0.9600
C4—C5	1.398 (2)	C18—H18C	0.9600
C4—H4	0.9300	C20—C21	1.441 (12)
C5—C6	1.384 (2)	C20—H20A	0.9700
C5—C19	1.480 (2)	C20—H20B	0.9700
C6—C7	1.387 (2)	C21—H21A	0.9600
C6—H6	0.9300	C21—H21B	0.9600
C8—C9	1.381 (3)	C21—H21C	0.9600
C8—C13	1.392 (2)	C21A—C20A	1.440 (13)
C9—C10	1.381 (3)	C21A—H21D	0.9600
C9—H9	0.9300	C21A—H21E	0.9600
C10—C11	1.370 (3)	C21A—H21F	0.9600
C10—H10	0.9300	C20A—H20C	0.9700
C11—C12	1.373 (3)	C20A—H20D	0.9700
C11—H11	0.9300		
			
C13—O1—C14	118.20 (15)	N2—C15—C17	111.19 (16)
C19—O3—C20A	118.4 (17)	N2—C15—C16	111.76 (15)
C19—O3—C20	116.7 (6)	C17—C15—C16	113.08 (17)
C1—N1—C7	104.53 (13)	N2—C15—H15	106.8
C1—N2—C2	106.11 (12)	C17—C15—H15	106.8
C1—N2—C15	125.83 (13)	C16—C15—H15	106.8
C2—N2—C15	127.75 (13)	C15—C16—H16A	109.5
N1—C1—N2	113.72 (13)	C15—C16—H16B	109.5
N1—C1—C8	122.94 (14)	H16A—C16—H16B	109.5
N2—C1—C8	123.27 (14)	C15—C16—H16C	109.5
N2—C2—C3	133.33 (15)	H16A—C16—H16C	109.5
N2—C2—C7	105.16 (13)	H16B—C16—H16C	109.5
C3—C2—C7	121.52 (15)	C18—C17—C15	113.38 (19)
C4—C3—C2	117.02 (15)	C18—C17—H17A	108.9
C4—C3—H3	121.5	C15—C17—H17A	108.9
C2—C3—H3	121.5	C18—C17—H17B	108.9
C3—C4—C5	122.30 (15)	C15—C17—H17B	108.9
C3—C4—H4	118.8	H17A—C17—H17B	107.7
C5—C4—H4	118.8	C17—C18—H18A	109.5
C6—C5—C4	120.35 (15)	C17—C18—H18B	109.5
C6—C5—C19	121.18 (16)	H18A—C18—H18B	109.5
C4—C5—C19	118.47 (15)	C17—C18—H18C	109.5
C5—C6—C7	118.24 (15)	H18A—C18—H18C	109.5
C5—C6—H6	120.9	H18B—C18—H18C	109.5
C7—C6—H6	120.9	O2—C19—O3	122.81 (18)
C6—C7—N1	128.98 (15)	O2—C19—C5	124.93 (19)
C6—C7—C2	120.55 (14)	O3—C19—C5	112.26 (15)
N1—C7—C2	110.46 (14)	C21—C20—O3	106.8 (8)
C9—C8—C13	119.04 (15)	C21—C20—H20A	110.4
C9—C8—C1	119.08 (16)	O3—C20—H20A	110.4
C13—C8—C1	121.81 (16)	C21—C20—H20B	110.4
C10—C9—C8	120.85 (19)	O3—C20—H20B	110.4
C10—C9—H9	119.6	H20A—C20—H20B	108.6
C8—C9—H9	119.6	C20—C21—H21A	109.5
C11—C10—C9	119.33 (19)	C20—C21—H21B	109.5
C11—C10—H10	120.3	H21A—C21—H21B	109.5
C9—C10—H10	120.3	C20—C21—H21C	109.5
C10—C11—C12	121.27 (17)	H21A—C21—H21C	109.5
C10—C11—H11	119.4	H21B—C21—H21C	109.5
C12—C11—H11	119.4	C20A—C21A—H21D	109.5
C11—C12—C13	119.38 (18)	C20A—C21A—H21E	109.5
C11—C12—H12	120.3	H21D—C21A—H21E	109.5
C13—C12—H12	120.3	C20A—C21A—H21F	109.5
O1—C13—C12	124.40 (17)	H21D—C21A—H21F	109.5
O1—C13—C8	115.49 (14)	H21E—C21A—H21F	109.5
C12—C13—C8	120.11 (17)	C21A—C20A—O3	101.4 (11)
O1—C14—H14A	109.5	C21A—C20A—H20C	111.5
O1—C14—H14B	109.5	O3—C20A—H20C	111.5
H14A—C14—H14B	109.5	C21A—C20A—H20D	111.5
O1—C14—H14C	109.5	O3—C20A—H20D	111.5
H14A—C14—H14C	109.5	H20C—C20A—H20D	109.3
H14B—C14—H14C	109.5		
			
C7—N1—C1—N2	0.4 (2)	C1—C8—C9—C10	−178.21 (17)
C7—N1—C1—C8	−176.62 (16)	C8—C9—C10—C11	0.0 (3)
C2—N2—C1—N1	−1.2 (2)	C9—C10—C11—C12	0.8 (3)
C15—N2—C1—N1	−175.22 (16)	C10—C11—C12—C13	−0.5 (3)
C2—N2—C1—C8	175.86 (16)	C14—O1—C13—C12	−10.5 (3)
C15—N2—C1—C8	1.8 (3)	C14—O1—C13—C8	169.60 (17)
C1—N2—C2—C3	−178.75 (18)	C11—C12—C13—O1	179.54 (17)
C15—N2—C2—C3	−4.9 (3)	C11—C12—C13—C8	−0.6 (3)
C1—N2—C2—C7	1.37 (18)	C9—C8—C13—O1	−178.76 (16)
C15—N2—C2—C7	175.26 (16)	C1—C8—C13—O1	−1.7 (2)
N2—C2—C3—C4	180.00 (18)	C9—C8—C13—C12	1.3 (3)
C7—C2—C3—C4	−0.1 (3)	C1—C8—C13—C12	178.40 (16)
C2—C3—C4—C5	1.5 (3)	C1—N2—C15—C17	111.1 (2)
C3—C4—C5—C6	−1.5 (3)	C2—N2—C15—C17	−61.7 (2)
C3—C4—C5—C19	177.90 (17)	C1—N2—C15—C16	−121.50 (19)
C4—C5—C6—C7	0.2 (3)	C2—N2—C15—C16	65.7 (2)
C19—C5—C6—C7	−179.26 (17)	N2—C15—C17—C18	−55.0 (2)
C5—C6—C7—N1	−178.97 (17)	C16—C15—C17—C18	178.28 (19)
C5—C6—C7—C2	1.2 (3)	C20A—O3—C19—O2	−13.3 (18)
C1—N1—C7—C6	−179.38 (19)	C20—O3—C19—O2	1.3 (5)
C1—N1—C7—C2	0.5 (2)	C20A—O3—C19—C5	167.2 (17)
N2—C2—C7—C6	178.70 (16)	C20—O3—C19—C5	−178.2 (5)
C3—C2—C7—C6	−1.2 (3)	C6—C5—C19—O2	−172.8 (2)
N2—C2—C7—N1	−1.19 (19)	C4—C5—C19—O2	7.8 (3)
C3—C2—C7—N1	178.91 (16)	C6—C5—C19—O3	6.7 (3)
N1—C1—C8—C9	65.8 (2)	C4—C5—C19—O3	−172.74 (17)
N2—C1—C8—C9	−110.9 (2)	C19—O3—C20—C21	172.8 (5)
N1—C1—C8—C13	−111.2 (2)	C20A—O3—C20—C21	−86 (10)
N2—C1—C8—C13	72.0 (2)	C19—O3—C20A—C21A	119 (2)
C13—C8—C9—C10	−1.1 (3)	C20—O3—C20A—C21A	33 (6)

### Hydrogen-bond geometry (Å, °)

**Table:**

*Cg* is the centroid of the N1/N2/C1/C2/C7 ring.

**Table:**

*D*—H···*A*	*D*—H	H···*A*	*D*···*A*	*D*—H···*A*
C12—H12···N1^i^	0.93	2.56	3.471 (2)	165
C20A—H20C···Cg^ii^	0.97	2.90	3.71 (4)	141

Symmetry codes: (i) −*x*+1/2, *y*−1/2, −*z*+3/2; (ii) −*x*, −*y*+2, −*z*+2.
